# Plasticity in Vulnerability to Cavitation of *Pinus canariensis* Occurs Only at the Driest End of an Aridity Gradient

**DOI:** 10.3389/fpls.2016.00769

**Published:** 2016-06-03

**Authors:** Rosana López, Francisco J. Cano, Brendan Choat, Hervé Cochard, Luis Gil

**Affiliations:** ^1^Forest Genetics and Physiology Research Group, Sistemas y Recursos Naturales, School of Forest Engineering, Technical University of MadridMadrid, Spain; ^2^Hawkesbury Institute for the Environment, University of Western SydneyRichmond, NSW, Australia; ^3^PIAF, INRA, Université Clermont AuvergneClermont-Ferrand, France

**Keywords:** vulnerability to cavitation, hydraulic conductivity, drought, phenotypic plasticity, genetic differentiation, provenance trials, *Pinus canariensis*

## Abstract

Water availability has been considered one of the crucial drivers of species distribution. However, the increasing of temperatures and more frequent water shortages could overcome the ability of long-lived species to cope with rapidly changing conditions. Growth and survival of natural populations adapted to a given site, transferred and tested in other environments as part of provenance trials, can be interpreted as a simulation of ambient changes at the original location. We compare the intraspecific variation and the relative contribution of plasticity to adaptation of key functional traits related to drought resistance: vulnerability to cavitation, efficiency of the xylem to conduct water and biomass allocation. We use six populations of Canary Island pine growing in three provenance trials (wet, dry, and xeric). We found that the variability for hydraulic traits was largely due to phenotypic plasticity, whereas, genetic variation was limited and almost restricted to hydraulic safety traits and survival. Trees responded to an increase in climate dryness by lowering growth, and increasing leaf-specific hydraulic conductivity by means of increasing the Huber value. Vulnerability to cavitation only showed a plastic response in the driest provenance trial located in the ecological limit of the species. This trait was more tightly correlated with annual precipitation, drought length, and temperature oscillation at the origin of the populations than hydraulic efficiency or the Huber value. Vulnerability to cavitation was directly related to survival in the dry and the xeric provenance trials, illustrating its importance in determining drought resistance. In a new climatic scenario where more frequent and intense droughts are predicted, the magnitude of extreme events together with the fact that plasticity of cavitation resistance is only shown in the very dry limit of the species could hamper the capacity to adapt and buffer against environmental changes of some populations growing in dry locations.

## Introduction

Water availability is a crucial driver of species distribution (Ramírez-Valiente et al., 2009). However, the increasing temperatures and more frequent water shortages associated with global climate change could overcome the ability of long-lived species to cope with rapidly changing conditions (Hoffmann and Sgrò, 2011; Choat et al., 2012). Ecosystem responses to these new climatic scenarios will include the interrelated processes of evolutionary change, shifts in geographic range and extinction of some populations (Nicotra et al., 2010). The relative role of each process is far from clear and will depend on how species and populations acclimate their structure and function (i.e., phenotypic plasticity) or adapt through natural selection. Another factor that could increase selective pressures in long-lived species is the decrease in gene flow due to severe habitat fragmentation (López de Heredia et al., 2010). This may lead to decreases genetic diversity, limitations on the ecological benefits of plasticity, and decoupling of climate and local adaptation (Jump and Peñuelas, 2005). Taken together these factors would result in increased vulnerability to extreme climatic events and to a higher risk of mortality of trees. This is particularly important for populations in the southernmost locations in the northern hemisphere which may become extinct if they are not able to adapt or migrate. Thus, studies that quantify the ability of species to maintain their fitness and plasticity of key traits should be a priority in management and conservation programs.

Survival and growth of natural populations adapted to a given location, transferred and tested in other environments as part of provenance trials or common garden tests, can be interpreted as a simulation of ambient changes at the original location and are also valuable tools to separate the genetic component of adaptation from phenotypic plasticity. Although, provenance trials have been intensively established during the last 250 years, they have been mainly used for productive purposes avoiding sites with low fertility or at the ecological limits of the species (Mátyás, 2002). Few studies have been implemented under conditions of severe change where populations are reaching their tolerance limits. In such marginal situations, the effectiveness of adjustment through natural selection is limited and mass mortality may occur. In fact, in recent years an overall increased global frequency in reported drought-related mortality events (Allen et al., 2010) has evidenced the increasing vulnerability to forest dieback.

Hydraulic failure due to xylem embolism is broadly accepted as a key factor of drought-induced mortality, whether directly rupturing the water continuum from soil to leaves or via carbon starvation due to prolonged stomatal closure (Sala et al., 2010). The importance of changes in the plant conducting system in response to drought has been highlighted in order to maximize water uptake and reduce hydraulic failure (Sperry et al., 2002; Ladjal et al., 2005; Brodribb and Cochard, 2009). Hydraulic traits thus play a crucial role in adaptation and can be used to predict the future resilience of forested ecosystems.

Plants differ widely in their vulnerability to drought-induced cavitation and the responses to drought are species-specific and depend on a tree’s hydraulic strategy (Bréda et al., 2006). Overall, conifers are more resistant to cavitation than angiosperms but less efficient in water transport (Maherali et al., 2004). The xylem of conifers, cheap to maintain but less efficient than broadleaved xylem, may confer a competitive advantage in low resource environments where photosynthesis is limited and water availability scarce (Hacke and Sperry, 2001). Despite the homogeneity of xylem structure, where tracheids make up almost 90% and the remainder is axial and ray parenchyma along with resin ducts in certain species (Plomion et al., 2001), striking interspecific variation in cavitation resistance has been reported (Piñol and Sala, 2000; Maherali et al., 2004; Martínez-Vilalta et al., 2004; Brodribb and Cochard, 2009; Delzon et al., 2010; Bouche et al., 2014). However, information about variation within species, and to what extent genotypes exhibit plasticity in hydraulic traits, remains scarce and very few studies have reported quantitative relationships between survival or growth and resistance to xylem embolism (Tyree et al., 2002; Brodribb and Cochard, 2009; Barigah et al., 2013; Urli et al., 2013). The study of such phenotypic variation is critical for both the development of a general understanding, and predicting plant responses to climate change. In this context, provenance trials are valuable tools to separate the effects of genetics and acclimation on phenotypic variation.

*Pinus canariensis* is endemic to the Canary Islands. Despite this restricted distribution area, volcanic destruction and successive fragmentation of populations, erosional activities and the influence of the humid Trade winds and the dry Saharan winds have created extremely diverse habitats that may exert varying selective pressures (Emerson, 2002). Current environmental conditions are very different from those in which this species evolved under a much wetter climate even during the late Holocene (de Nascimento et al., 2009). This species is therefore a good model to compare the intraspecific variation and the relative contribution of plasticity to adaptation of key functional traits related to drought resistance: vulnerability to cavitation, efficiency of the xylem to conduct water and biomass allocation. We use six populations of Canary Island pine growing in three provenance trials with contrasted climatic conditions: a wet location influenced by the Trade winds, a dry site in the leeward slopes of the Teide volcano and the most xeric site, in the very edge of the distribution of the species.

## Materials and Methods

### Provenance Trial Experiments

**FIGURE 1:**
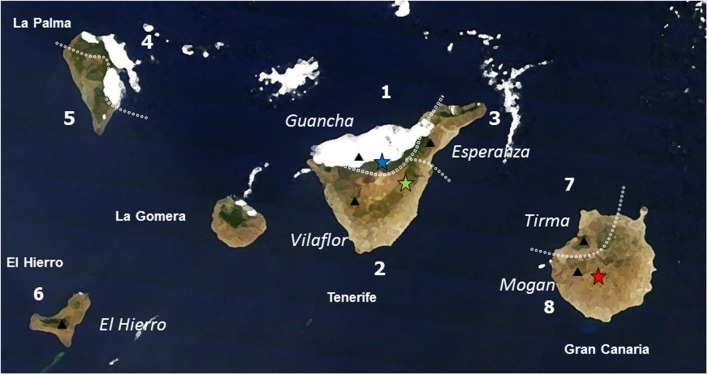
**Location of the sampled population (black triangles, names in italics) of *Pinus canariensis* and provenance trials (blue star: wet site; green star: dry site; red star: xeric site) in the western Canary Islands (names of the island in bold).** Dotted lines are the limits of the ecological regions (numbered) described in Climent et al. (2004, 2006). Note the influence of the humid Trade Winds.

We selected trees from six populations of *P. canariensis* growing in three provenance trials in the Canary Islands with contrasted climatic conditions. The most humid provenance trial (wet site onward) is influenced by the Trade winds which can even double the annual precipitation due to fog (795 mm MAP; **Figure 1**). This wet site and the dry site (460 mm MAP) have similar soils and temperatures but differ sharply in water availability and drought period (**Table 1**). Finally, the xeric site, located in the dryer limit of distribution of the species, combines an arid environment (320 mm MAP and the periodic gusts of the extreme dry Saharan wind) with very compact and stony soil.

**Table 1:** Climate characterization of the six populations of *Pinus canariensis* and the three provenance trials included in this study.

Ecological region	Population	Elevation	MAP (mm)	T (°C)	Tr (°C)	Dp (months)	ETo (mm/d)			
							sp	sum	aut	win
1	Guancha	700	939.9	12.7	14.4	3.6	3.56	4.41	2.34	2.39
2	Vilaflor	1900	505	13.2	22.2	5.36	4.15	4.77	2.73	2.84
3	Esperanza	1100	629.7	14.7	17.4	4.79	3.74	4.47	2.41	2.50
6	El Hierro	1000	450.1	16.4	15.7	6.66	4.2	4.80	2.76	2.90
7	Tirma	850	379.6	18	20.6	6.83	4.37	4.84	2.85	2.99
8	Mogán	900	334.7	17.6	21.9	7.52	4.37	4.84	3.00	3.00
Provenance trials
1	Wet	1575	795	14.3	21.1	4.07	3.70	4.46	2.39	2.47
2	Dry	1720	460	11.4	21.0	4.82	4.21	4.80	2.77	2.89
8	Xeric	1259	320	17.8	20.3	7.68	4.36	4.90	3.00	3.01

*MAP: annual precipitation, T: mean annual temperature, Tr: annual temperature range, Dp: drought period; ETo: Evapotranspiration calculated with Penman-Monteith equation (sp: spring; sum: summer; aut: autumn; win: winter).*

The six populations included in this study, out of 21 growing in the provenance trials (more details about the establishment and populations included in these provenance trials can be found in López et al., 2007) were selected to cover both the range of the climatic envelope of the species and populations with different ages based on the chronostratigraphy of the substrate where they occur (more details in López de Heredia et al., 2014).

Survival, height and basal diameter were measured during the first 6 years after the establishment of the provenance trials. After this period survival rates were stable.

### Vulnerability to Cavitation

To evaluate the phenotypic plasticity of vulnerability to cavitation and hydraulic efficiency in this species we have used data from the wet and the xeric provenance trials already published in López et al. (2013). We have completed these data with unpublished measurements of the dry provenance trial. We describe in short the plant material and the methods used.

One branch exposed to the sun, longer than 40 cm and with a maximum diameter of 1 cm was sampled from 8 to 14 trees per population in each provenance trial. In the wet and xeric site, we sampled branches in 2010, when trees were 11 years-old and sampled branches corresponded to the previous year growth unit in the wet site and to the last 2–3 years units in the wet site. Needles were removed and branches were wrapped in a black plastic bag with moist paper towels to prevent dehydration. In the dry site, branches were collected 2 years later following the same procedure.

Vulnerability curves of branches from the wet and the xeric sites were constructed with the Cavitron technique (Cochard et al., 2005). For a detailed description of the methods see López et al. (2013). The vulnerability curves of branches from the dry site were constructed with the standard centrifuge method. Both, the Cavitron and the static centrifuge have shown similar results in conifer species (Li et al., 2008). Branches were trimmed under water and then both ends were shaved to a final length of 14 cm. The initial hydraulic conductance (*k*i, mol s^-1^ MPa^-1^) was measured using a XYL’EM device (Xylem Embolism Meter, Bronkhorst, Montigny les Cormeilles, France) at low pressure (4–5 × 10^-3^ MPa), perfusing the samples with the same solution described above. After measuring *k*i, branches were spun in a centrifuge for 5 min at increasing pressure steps to achieve negative xylem pressures (Alder et al., 1997). After each step, *k*h was measured with the XYL’EM.

The percentage loss of hydraulic conductivity (PLC) was estimated by the step-by-step decrease of *k*h with regard to *k*i as: PLC = 100 × (1 – *k*h/*k*i). The observed curve was fitted to a logistic function (Pammenter and Vander Willigen, 1998):

$$PLC\left(\%\right)=100/\left[1+\exp\left(s/25\left(P-P_{50}\right)\right)\right]$$

where P_50_ represents the value of Ψ at which 50% of hydraulic conductivity is lost and *s* is the slope of the VC at P_50_. Finally, estimates of xylem water potentials at the beginning of xylem embolism (P_12_) and full embolism (P_88_) were calculated following Domec and Gartner (2001):

$$P_{12}=P_{50}+50/s$$

$$P_{88}=P_{50}-50/s.$$

### Hydraulic Conductivity

Xylem specific hydraulic conductivity (*K*s) was assessed dividing *k*i by the sapwood area in the middle of the branch (*A*s) and multiplying by sample length. Leaf specific hydraulic conductivity (*K*l) was calculated as the conductivity per unit of projected leaf area (*A*l). All leaves distal to the branch section used for constructing the vulnerability curve were collected. The projected area of 12 leaves per sample was obtained with a scanner and analyzed with WinFOLIA (Regent Instruments, Inc., Canada). Then they were dried at 60°C for 3 days to determine leaf dry mass and leaf mass per area (LMA). The rest of the leaves were dried as previously described and total leaf area was calculated dividing the total needle mass by LMA. The Huber value (HV), i.e., the ratio of the sapwood cross sectional (*A*s) to the distal leaf area supported (*A*l), was also calculated.

### Statistical Analysis

Differences among populations and between the three provenance trials for growth, biomass allocation, vulnerability to cavitation, and hydraulic conductivity were assessed using a general linear model (GLM) with the fixed factors provenance trial, population and their interaction. Survival was analyzed with a linear logistic model with the same factors considering a binomial distribution of the data and a logit function. The percentage of variation explained by each factor was calculated with the variance components, assuming all the factors were random. We assumed that phenotypic plasticity occurred if the effect of the environment (provenance trial) in the GLM was significant and that genotypes (populations) differed in plasticity if the interaction population × provenance trial was significant.

Correlations between traits were evaluated by calculating Pearson’s coefficient on the population Best Linear Unbiased Estimator (BLUE). In addition, Spearman’s correlation coefficients were determined between the climatic conditions at origin and the BLUEs of hydraulic and growth traits of each population. All analyses were performed using STATISTICA v. 7.0 (StatSoft, Inc.).

## Results

### Phenotypic Plasticity and Genetic Variation in Survival, Growth, and Hydraulic Traits

**FIGURE 2:**
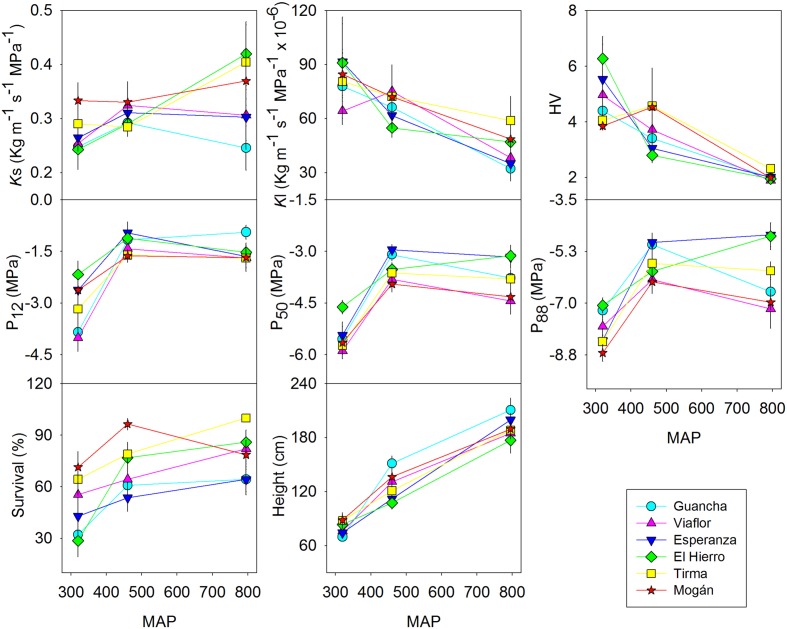
**Reaction norms of six populations of *P. canariensis* planted in three provenance trials (wet, dry and xeric) for xylem specific conductivity (*K*s), leaf specific conductivity (*K*l), Huber value (HV), xylem potential at 12% (P_12_), 50% (P_50_), and 88% (P_88_) loss of conductivity, survival and height.** Error bars represent the standard error.

As expected, survival and growth were higher in the wet provenance trial and lower in the xeric one. In this last site we found the most striking differences between provenances: those from drier locations survived better than those from more mesic habitats. This pattern was particularly striking in the xeric site although one of the arid provenances El Hierro, exhibited higher than expected mortality (**Figure 2**). We did not find any significant difference in height or basal diameter among populations but a substantial phenotypic plasticity (**Table 2**) and almost a linear increment with MAP (**Figure 2**). Six years after planting, mean height in the wet provenance trial reached almost 2 meters, in the dry 126 cm while in the xeric average height was 80 cm (**Figure 2**).

**Table 2:** Percentage of the explained variation and significance values (^∗^*p* < 0.05; ^∗∗^*p* < 0.01; ^∗∗∗^*p* < 0.001) due to provenance trial, population and the interaction provenance trial by population for six populations of *P. canariensis* growing in three provenance trials.

Trait	Provenance trial	Population	Provenance Trial × Population
Survival	21.87 ^∗∗∗^	5.08 ^∗∗∗^	2.21
H	73.11 ^∗∗∗^	0.61	0.77
Db	52.70 ^∗∗∗^	1.61	1.22
*K*s	3.90 ^∗^	1.69	0.22
*K*l	17.71 ^∗∗∗^	0	0
HV	29.03 ^∗∗∗^	0	5.70
P_12_	41.29 ^∗∗∗^	6.01 ^∗^	1.33
P_50_	59.86 ^∗∗∗^	7.74 ^∗∗∗^	1.82
P_88_	43.83 ^∗∗∗^	8.24 ^∗∗∗^	7.47 ^∗∗^

*H: height 6 years after planting; Db: basal diameter 6 years after planting; Ks: specific hydraulic conductivity; Kl: leaf specific hydraulic conductivity; HV: Huber value; P_12_, P_50_, P_88_: xylem water potential at 12, 50, and 88% loss of conductivity respectively*.

Trees growing at the wet provenance trial had a more permeable xylem, i.e., higher *K*s. This was evident in two of the six populations (Tirma and El Hierro, **Figure 2**, Supplementary Figure S1). On the contrary, the leaf area supplied by a given xylem area, *K*l, was significantly higher in the xeric site due to a Huber value twice as higher and decreased almost linearly with MAP (**Figure 2**, Supplementary Figure S1). In this provenance trial, only current year leaves persist in the trees, whereas in the wet and dry sites it is common to find more than three cohorts of leaves. Most of the variability of hydraulic conductivity traits remained within populations rather than between populations or between sites (**Table 2**).

In the wet and dry provenance trials, embolism began at similar water potential, i.e., similar P_12_, average -1.4 MPa, but followed at different rates in each population, as shown by different slope of the vulnerability curves (Supplementary Figure S1), resulting in similar values of P_50_ and P_88_ in both sites for a specific population, with significant differences between populations (**Figure 2**, Supplementary Figure S1). For instance, P_50_ varied from -3.1 to -4.1 MPa for Esperanza and Mogan, respectively. In the xeric provenance trial, P_12_ dropped to -3.0 MPa and P_50_ ranged from -4.6 MPa in El Hierro to -5.9 MPa in Vilaflor. Values of water potential at full embolism, P_88_, varied between c. -6 MPa in the wet and dry provenance trials and almost -8 MPa in the xeric one (**Figure 2**). On average, 8% of the observed phenotypic variation in P_50_ and P_88_ was due to between-population differences whereas 60 and 44% were due to the provenance trial, respectively (**Table 2**). Genotype × environment (i.e., population × provenance trial) interaction was only significant for P_88_ (7.5% of the variance), reflecting the similar plasticity of all populations for hydraulic safety traits.

It is worth mentioning that we did not flush the branches before constructing the vulnerability curves. To discard the effect of variations in native state levels of embolism on the curves, we plotted changes in *K*s with increasing xylem tension (Supplementary Figure S1). Initial values of *K*s for a given provenance across sites were only significantly higher in the wet provenance trial for El Hierro and Tirma and it fell more steeply than in the other two trials but never converged. Moreover, we did not find any correlation between changes in *K*s and changes in P_50_ across sites for any provenance (*p* > 0.1; data non-shown). For the most resistant provenances the slope was steeper between -2.5 and -5.5 MPa in the wet and the dry sites and 1 MPa lower in the xeric site whereas for the others, the steepest fall in *K*s was found between -2 and -4.5 MPa in the wet and dry site and also 1 MPa lower in the xeric site (Supplementary Figure S1). These results suggested that both the vulnerability curves, expressed as PLC or as *K*s, showed the same information. Finally, when the loss of conductivity was expressed as *K*l, we found a remarkable difference among sites, even between the wet and the dry provenance trials for some provenances, despite the almost overlapping vulnerability curves expressed as PLC. These differences could have been overestimated at high xylem tensions as trees adjust their foliar area to water availability.

### Relationship between Hydraulic Properties, Survival, Growth, and Climate

**FIGURE 3:**
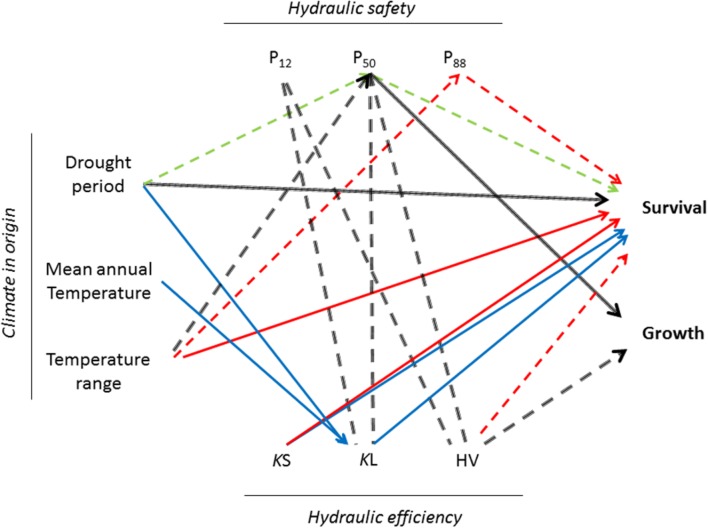
**Pairwise correlations between climate in the origin, hydraulic traits and survival and growth of six populations of Canary Island pine planted in three provenance trials.** Only significant relationships (*p* < 0.05) are depicted. Dashed lines indicate negative relationships. In black overall correlations (pooling data from the three provenance trials). Blue: wet provenance trial, Green: dry provenance trial, Red: xeric provenance trial. *K*s: xylem specific conductivity, *K*l: leaf specific conductivity, HV: Huber value (HV), P_12_, P_50_, P_88_ xylem potential at 12, 50, and 88% loss of conductivity, respectively.

Pooling data from the three sites, taller trees were related to lower HV (*r* = -0.45, *p* < 0.001) and less negative P_50_, P_12_, and P_88_ (average *r* = 0.35, *p* < 0.001). We observed weak correlations between HV and K_l_ with P_12_ (*r* = -0.23, *p* < 0.01; *r* = -0.15, *p* < 0.05) and P_50_ (*r* = -0.20, *p* < 0.01; *r* = -0.15, *p* < 0.05). However, none of these relationships were found within provenance trials (**Figure 3**).

Aridity at the origin of the populations influenced survival and vulnerability to cavitation in the dry and the xeric sites. Plants from locations with less precipitation and a longer drought period survived better (ρ = -0.94) and constructed a safer xylem (ρ = -0.77). Moreover, survival in the dry provenance trial was strongly correlated with P_50_ (ρ = -0.83) and in the xeric with P_88_ (ρ = -0.94). On the contrary, in the wet provenance trial the length of the drought period was not related with any parameter derived from the vulnerability curve but with *K*l (ρ = -0.94). Here, survival was more related with the efficiency of water transport, *K*s (ρ = 0.89) and *K*l (ρ = 0.83). Finally, we found a negative trend between temperature range and P_50_ in the three provenance trials (**Figure 3**).

## Discussion

### Plasticity in the Plant Hydraulic System

Despite pronounced site-of-origin differences in precipitation and temperature, *P. canariensis* populations expressed broadly similar patterns of growth and functional plasticity in three contrasting habitats (**Figure 2**). Hydraulic traits in plants from all six populations exhibited acclimation to drier and warmer conditions with more cavitation resistant xylem, increased leaf specific conductivity and structurally increased Huber value by dramatically reduced the leaf area.

The evolution of plasticity in key functional traits may determine an organism’s ability to establish (Schlichting, 1986), colonize new environments (Matesanz et al., 2012) and persist in highly variable environments or over broad niches if plasticity increases that organisms’ fitness. In this sense, the importance of changes in the plant conducting system in response to drought has been highlighted in order to maximize water uptake and reduce hydraulic failure (Sperry et al., 2002; Brodribb and Cochard, 2009). Within a single species, the variance of hydraulic traits can have a major impact on population demographics and responses to changes in climate extremes (Anderegg et al., 2013). One general trend found in interspecific comparisons is that taxa growing in drier habitats tend to exhibit a safer xylem (Hacke et al., 2000; Pockman and Sperry, 2000; Maherali et al., 2004; Choat et al., 2012), although such studies may confound both genetic variation and phenotypic plasticity. The few studies quantifying intraspecific phenotypic plasticity for cavitation have reported lower values of P_50_ when plants of a given population grow in drier environments (Corcuera et al., 2011; Wortemann et al., 2011) or after water withholding (Aranda et al., 2015), but see Lamy et al. (2014) for an exception. Constructing a more cavitation-resistant xylem allow plants to maintain higher stomatal conductance despite increasing water stress in drier or warmer habitats. Interestingly, we only observed this trend in the xeric provenance trial. The vulnerability to cavitation curves in the wet and the dry provenance trials were very similar, despite remarkable variation in environmental conditions between both sites (Supplementary Figure S1). Therefore, it appears that only trees growing at the very edge of the distribution limit exhibit plasticity of cavitation resistance and with a shift only reported for this species, almost 2 MPa (López et al., 2013 and the present work), comparing with changes lower than 1 MPa found in beech and maritime pine (Corcuera et al., 2011; Wortemann et al., 2011; Aranda et al., 2015) or in populations of Canary Island pine from La Palma (López et al., 2013). In fact, cavitation resistance of Canary Island pines growing in the xeric provenance trial is one of the highest found in pines (Bouche et al., 2014), consistent with its pioneer behavior, its capacity to colonize bole volcanic soils, and with an extremely low capacity to retain water after eruptions.

Another key change in the hydraulic architecture entails the reduction of the water potential gradient for a given transpiration rate. Increasing *K*l may assist in maintaining xylem water potentials above the level that would trigger cavitation during high evaporative demand or low soil water availability (Choat et al., 2007). In the present study, the average *K*l in each provenance trial was in accordance with this trend and it increased gradually from the wet to the xeric one. The bulk of the variation in *K*l was driven by variation in HV rather than changes in *K*s as reflected by the differences in the curves in Supplementary Figure S1, indicating that shifts in branch sapwood:leaf area allocation influence more the response of the hydraulic capacity than changes in xylem permeability. Thus, in the dry provenance trial *P. canariensis* relied on higher *K*l to reduce water potential gradients and lower leaf area to decrease the water use, thus saving soil water, rather than depend upon greater resistance to cavitation during summer or prolonged drought periods. Changes in the ratio of conducting to transpiration tissues is common in isohydric species such as pines and more intraspecific phenotypic variability has been found in this trait that in cavitation resistance. Xeric populations of *P. ponderosa* have higher HV and *K*l than mesic populations (Maherali and DeLucia, 2000) but like in *P. canariensis*, presumably as a result of phenotypic plasticity rather than ecotypic differentiation (Maherali et al., 2002). In *P. sylvestris* drier populations also follow this pattern of branch carbon allocation (Martinez-Vilalta et al., 2009) whereas, *P. palustris* and *P. halepensis* decreased the HV in xeric habitats or under severe drought but allocated more carbon to root production increasing the root to leaf area ratio (Tognetti et al., 1997; Addington et al., 2006).

In a recent review focused on plasticity of cavitation resistance, Anderegg (2015) found a substantial spatial variation within species and even larger within population variability in P_50_. In accordance, Canary Island pine populations from the windward slopes of Tenerife and El Hierro were more vulnerable than populations from the leeward slopes of Tenerife and Gran Canaria. Although constant plasticity (i.e., similar plasticity of different populations), as shown for most traits in our study, reduces the strength of diversifying selection and can alter the impact of gene flow on local adaptation in heterogeneous environments (Crispo, 2008), volcanism and aridity could have exerted selective pressures strong enough in traits related to drought resistance as to counteract for the homogenizing effect of an extensive gene flow (López de Heredia et al., 2014). This seems to be the case of genetic variation in cavitation resistance and other drought adaptive traits at the leaf level: sclerophylly, osmotic adjustment and leaf anatomy (López et al., 2009, 2010, 2013) whereas for other such *K*l, *K*s or growth most of the variation resides within populations (López et al., 2013; the present study). On the other hand, some authors have suggested that stressful environments are the most likely to result in the expression of higher levels of variation (Schlichting, 2008) as differences in survival in our study among populations in the drier edge of the ecological niche for the species (**Figure 2**).

### Climate Drivers of Cavitation Resistance

Plant hydraulic architecture has evolved to changes in climate over evolutionary timescales. For instance, dry periods drove the adaptation of cavitation resistant xylem in *Cupressaceae* at multiple points in the past 30 million years (Pittermann et al., 2012). Evidence gathered across species from a wide range of habitats, including conifers and evergreen angiosperms have pointed to a positive correlation between vulnerability to cavitation and water availability (Maherali et al., 2004; Jacobsen et al., 2007). Although very valuable to assess general trends, phylogeny and other historical constraints such as glaciations can interfere with the trait-climate relationships when applying to lower scales such as species of the same family or intraspecific trends. A good alternative for understanding how precipitation patterns influence xylem structure and function is to consider species that occur across wide moisture gradients. Despite its restricted distribution area, *P. canariensis* is a good model because it inhabits a wide range of climatic conditions and it is under strong selection pressures. Our results showed that cavitation resistance was more tightly correlated with annual precipitation, drought length, and temperature oscillation at the origin of the populations than hydraulic efficiency or the HV and also directly related to survival in the dry and the xeric provenance trials, illustrating its importance in determining drought resistance. As expected, the drier populations were the more resistant to cavitation and the mesic ones the more susceptible. This pattern is in accordance with several studies showing that populations from drier environments are less vulnerable than those from wetter environments (Alder et al., 1996; Mencuccini and Comstock, 1997; Sparks and Black, 1999; Choat et al., 2007; Aranda et al., 2015) and with less drought damage in subraces of *Eucalyptus globulus* originating from areas with more temperature seasonality (Dutkowski and Potts, 2012).

### Xylem Resistance to Cavitation Is an Adaptive Trait but What Is the Cost?

A growing literature over the last years has linked cavitation resistance to life-story traits, demography and crown desiccation after drought and fire (Pratt et al., 2007, 2008; Nardini et al., 2013). The water potential at incipient cavitation, P_12_, is linked in many species to stomatal closure (Brodribb et al., 2003; Meinzer et al., 2009; Nolf et al., 2015) and carbon assimilation (Aranda et al., 2015); P_50_ has emerged as an appropriate trait for modeling of forest die-off on a local scale under climate changes scenarios (Nardini et al., 2013) and P_88_ seems to be the threshold to recover normal physiological function or resprout after severe drought episodes in angiosperms (Urli et al., 2013; Li et al., 2016). Moreover, the straight correlation in our study between survival in dry and xeric environments and P_50_ and P_88_ highlighted the adaptive value of cavitation resistance. However, increased cavitation resistance is often thought to come at the expense of reduced plant growth (Cochard et al., 2007). Such a tradeoff is expected if increased cavitation resistance lies in the necessity to build a denser wood, which in conifers is achieved with thicker tracheids, a feature supposed to be costly in terms of carbon allocation (Enquist et al., 1999). We found a clear trade-off between cavitation resistance and height when considering the three provenance trials (**Figure 3**) but within each one we could not detect differences in growth between populations, thus although both traits showed a strong response to water availability they could not be interrelated.

We also failed to detect a trade-off between hydraulic efficiency and safety at the tissue level within provenance trial but when pooling data *K*l and HV were negatively correlated with P_12_ and P_50_ (**Figure 3**). This counterintuitive correlation was mainly due to the nearly universal trend of decreasing *A*l with climatic dryness coupled with comparatively little changes in *K*s observed in *P. canariensis*: a 9 and 20% decreased of *K*s in the dry and the xeric provenance trial respectively regarding the wet provenance trial was offset by 82 and 139% increase in HV. Moreover, the lack of a safety-efficiency trade-off could be explained in gymnosperms because of decoupling in the anatomical traits that control it; hydraulic efficiency is related to most strongly tracheid lumen diameter whereas safety is controlled by the overlap between the torus and the pit aperture (Pittermann et al., 2010; Bouche et al., 2014).

## Conclusion

Vulnerability to cavitation appeared to be the key factor for survival and maintenance of a positive carbon balance for *P. canariensis* in xeric environments. In dry areas shifts in branch sapwood:leaf area allocation influence more the response of the hydraulic capacity than changes in xylem permeability or vulnerability to cavitation. Water limitation in the leeward slopes of the Canary Islands seems to have been a powerful agent of natural selection promoting local adaptation in this species, despite high levels of gene flow among populations. Nevertheless, in a new climatic scenario where more frequent and intense droughts are predicted, the magnitude of extreme events together with the fact that plasticity of vulnerability to cavitation is only shown in the very dry limit of the species could hamper the capacity to adapt and buffer against environmental changes of some populations growing in dry locations.

## Author Contributions

LG led the establishment of the provenance trials. RL, HC, and LG designed the experiment. RL and FC carried out the field and lab measurements. All authors contributed to interpret the results. RL drafted the manuscript. All authors read and approved the final manuscript.

## Conflict of Interest Statement

The authors declare that the research was conducted in the absence of any commercial or financial relationships that could be construed as a potential conflict of interest.
