# Supplementary material for: Plasticity in Vulnerability to Cavitation of Pinus canariensis Occurs Only at the Driest End of an Aridity Gradient
Source: Front Plant Sci. 2016 Jun 3;7:769. doi: 10.3389/fpls.2016.00769 (PMC4891331; doi:10.3389/fpls.2016.00769)
Supplement: Supplementary file 1 [file Data_Sheet_1.PDF]

Supplementary Material

Plasticity in vulnerability to cavitation of *Pinus canariensis* occurs only at the driest end of an aridity gradient.

Authors: Rosana López, Francisco Javier Cano, Brendan Choat, Hervé Cochard, Luis Gil

* **Correspondence:** Corresponding Author: rosana.lopez@upm.es

Supplementary Figure 1. Branch xylem vulnerability curves of six populations of *Pinus canariensis* planted in three provenance trials (blue: wet provenance trial, green: dry provenance trial and red: xeric provenance trial) expressed as percentage loss of conductivity (PLC, upper panels), loss of specific conductivity (*K*s, middle panels) and loss of leaf specific conductivity (*K*l, lower panels) at different xylem water potentials (Ψ_{xyl}). Error bars represent the standard error.

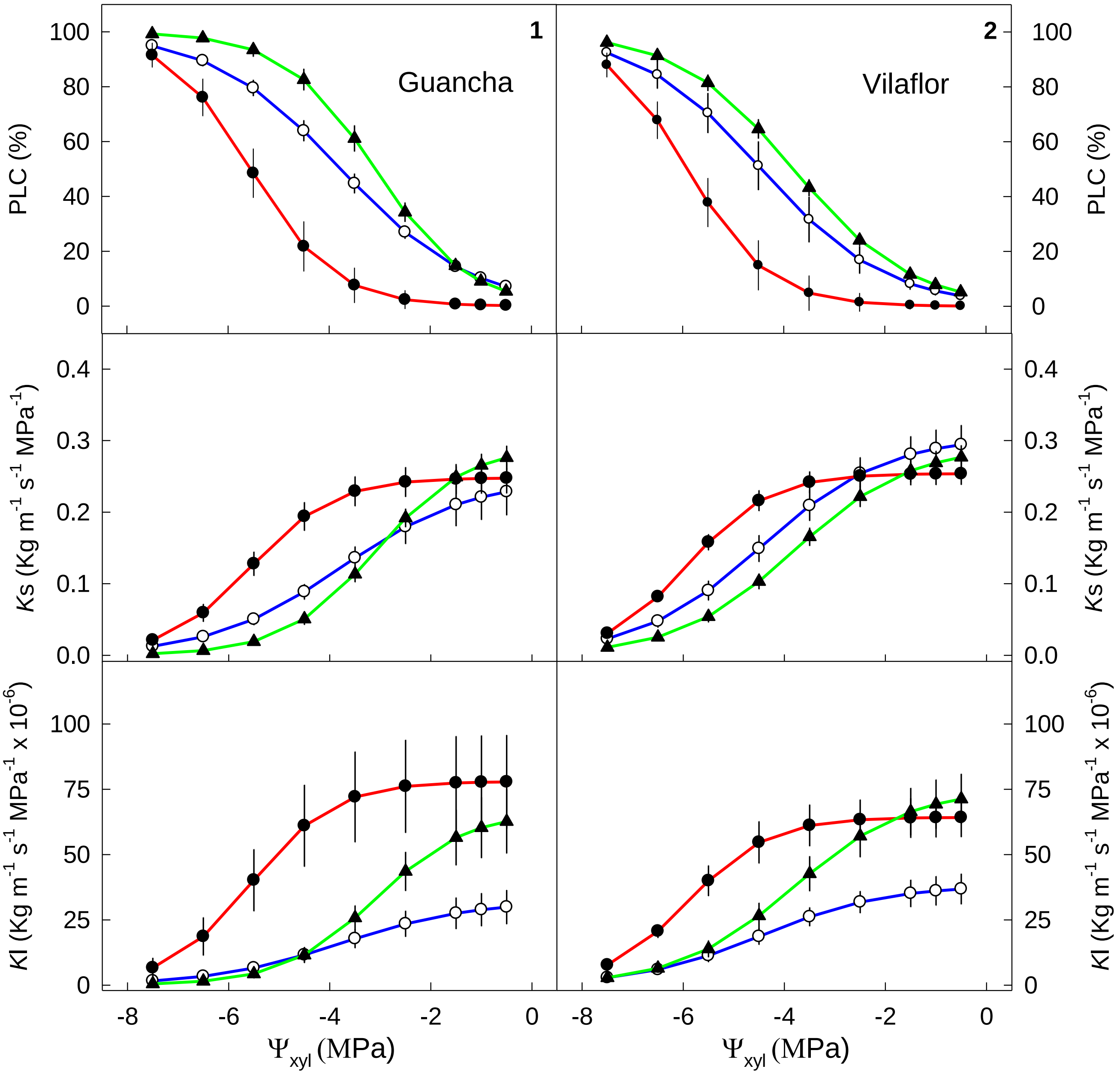

1
Guancha
2
Vilaflor
PLC (%)
0
20
40
60
80
100
Ks (Kg m-1 s-1 MPa-1)
0.0
0.1
0.2
0.3
0.4
Kl (Kg m-1 s-1 MPa-1 x 10-6)
0
25
50
75
100
-8
-6
-4
-2
0
Ψxyl (MPa)

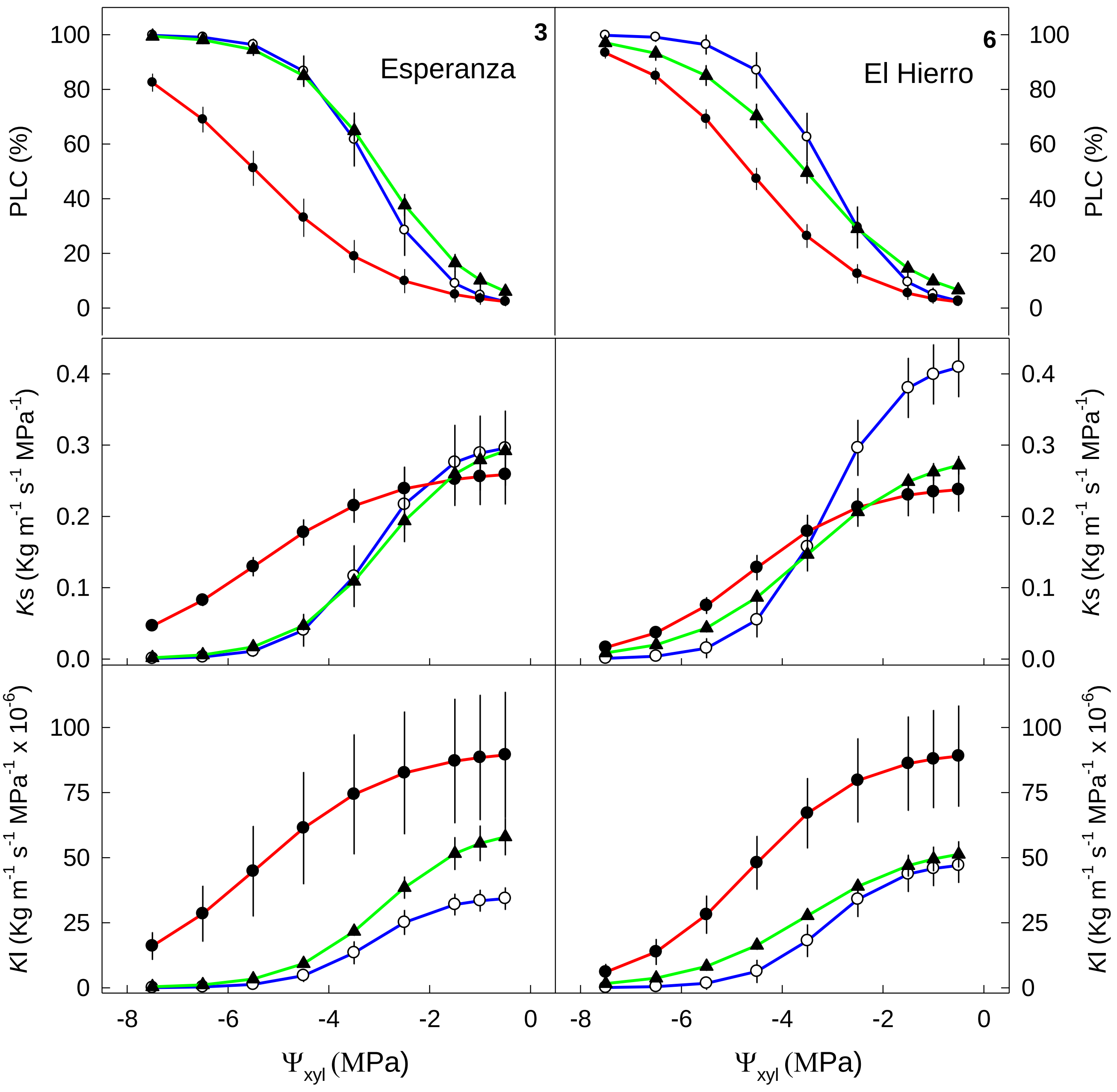
3
Esperanza
6
El Hierro
PLC (%)
Ks (Kg m-1 s-1 MPa-1)
Kl (Kg m-1 s-1 MPa-1 x 10-6)
Ψxyl (MPa)

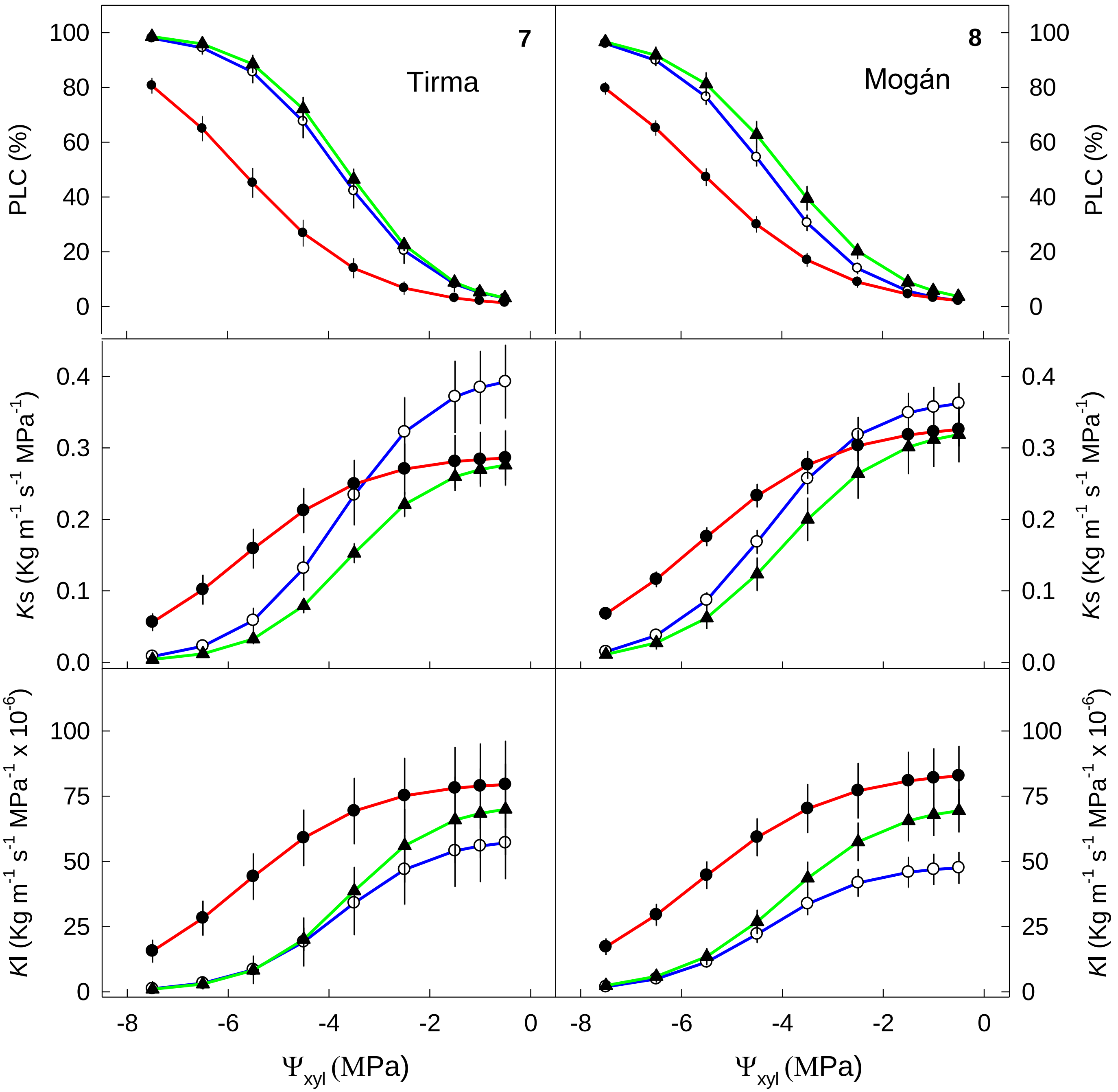
7
Tirma
8
Mogán
PLC (%)
Ks (Kg m-1 s-1 MPa-1)
Kl (Kg m-1 s-1 MPa-1 x 10-6)
Ψxyl (MPa)
